# Cancer Stem Cells—Key Players in Tumor Relapse

**DOI:** 10.3390/cancers13030376

**Published:** 2021-01-20

**Authors:** Monica Marzagalli, Fabrizio Fontana, Michela Raimondi, Patrizia Limonta

**Affiliations:** Department of Pharmacological and Biomolecular Sciences, University of Milan, via Balzaretti 9, 20133 Milano, Italy; fabrizio.fontana@unimi.it (F.F.); michela.raimondi@unimi.it (M.R.); patrizia.limonta@unimi.it (P.L.)

**Keywords:** cancer stem cells, drug resistance, molecular mechanisms, chemotherapy, radiation therapy, targeted therapies, immunotherapies

## Abstract

**Simple Summary:**

Cancer is one of the hardest pathologies to fight, being one of the main causes of death worldwide despite the constant development of novel therapeutic strategies. Therapeutic failure, followed by tumor relapse, might be explained by the existence of a subpopulation of cancer cells called cancer stem cells (CSCs). The survival advantage of CSCs relies on their ability to shape their phenotype against harmful conditions. This Review will summarize the molecular mechanisms exploited by CSCs in order to escape from different kind of therapies, shedding light on the potential novel CSC-specific targets for the development of innovative therapeutic approaches.

**Abstract:**

Tumor relapse and treatment failure are unfortunately common events for cancer patients, thus often rendering cancer an uncurable disease. Cancer stem cells (CSCs) are a subset of cancer cells endowed with tumor-initiating and self-renewal capacity, as well as with high adaptive abilities. Altogether, these features contribute to CSC survival after one or multiple therapeutic approaches, thus leading to treatment failure and tumor progression/relapse. Thus, elucidating the molecular mechanisms associated with stemness-driven resistance is crucial for the development of more effective drugs and durable responses. This review will highlight the mechanisms exploited by CSCs to overcome different therapeutic strategies, from chemo- and radiotherapies to targeted therapies and immunotherapies, shedding light on their plasticity as an insidious trait responsible for their adaptation/escape. Finally, novel CSC-specific approaches will be described, providing evidence of their preclinical and clinical applications.

## 1. Introduction: The Clinical Issue of Tumor Recurrence

Cancer still represents a major clinical issue and is one of the main causes of death worldwide. Despite the development of innovative therapeutic strategies and the improvement of diagnostic tools, cancer mortality rates remain high, with 18.1 million new cases and 9.6 million deaths occurred in 2018 [1,2]. Most patients must often cope with cancer recurrence after one or multiple treatment failures and this is due to the development of drug resistance [3]. The existence of cancer stem cells (CSCs) may explain this phenomenon, since the presence of CSCs markers have been generally considered a negative prognostic factor, and correlated with poor overall-free survival and poor disease-free survival within a variety of tumor types, such as ovarian cancer [4], head and neck squamous cell carcinoma [5], renal cell carcinoma [6], colorectal cancer [7], lung cancer [8], hepatocellular carcinoma [9]. Moreover, recent clinical and preclinical findings confirmed the relevance of CSC-specific pathways in cancer progression/relapse, as a result of drug resistance, in different cancers, such as ovarian cancer [10], glioblastoma [11,12], colorectal cancer [13], and leukemia [14]. Given these observations, this review will summarize the general features of CSCs, focusing on their crucial involvement in the resistance to different treatments.

## 2. General Features of CSCs

CSCs represent the most aggressive population of cells within the tumor mass and are usually defined as tumor initiating cells [15]. Characterizing feature of CSCs is their ability to maintain their undifferentiated state through self-renewal, coupled to a high differentiating potential, thus allowing the maintenance of a stem cell pool together with the generation of a heterogeneous progeny of differentiated tumor cells. Two different theories have been proposed to describe the role of CSCs: (1) in the hierarchical theory, CSCs are located at the top of the hierarchy and are able to generate every single clone of the tumor bulk, thus making them the root of the heterogeneous growing tumor; (2) in the stochastic theory, tumors originate from random mutations occurring in normal cells, additive mutations can then promote the acquisition of a stemness phenotype, thus every single cell within the tumor bulk has the potential to become a CSC [16,17,18]. However, as proposed in more recent models, these two theories are not mutually exclusive, and the plasticity of cancer cells seems to be crucial for the interconversion between stem and non-stem phenotypes. In this context, as a paradigm of cell plasticity, the epithelial-to-mesenchymal transition (EMT) process, as well as the reverse transition (mesenchymal-to-epithelial), has been extensively related to cancer progression [19,20]. However, in previous concepts, the EMT/MET has been considered as a binary process, whereas the emerging concept is that, between the EMT and the MET state, several phenotypic manifestations of a hybrid epithelial-mesenchymal (E/M) state are observed in different tumors [21], and have been strictly related to stemness features [22,23]. An elegant study by Pastushenko et al. shows that, in mouse models of lung cancer and squamous cell carcinomas, deletion of the protocadherin Fat1 is responsible for the acquisition of a hybrid E/M state by inducing YAP1 (a mesenchymal inducer) paralleled by the induction of SOX2 (an epithelial inducer). Interestingly, those factors are involved in the acquisition of stemness traits in cancer cells: indeed, Fat1-depleted cells had higher tumorigenic potential, and Fat1-KO mice showed high-spontaneous metastasis rates [24]. Moreover, preclinical and clinical evidences show the manifestation of the hybrid E/M phenotype in circulating tumor cells (CTCs), often travelling as clusters within the bloodstream, thus enhancing the metastatic potential and niching to secondary sites in breast cancer [25], colorectal cancer [26], and different other tumor types [27]. Importantly, CTCs have been demonstrated to show CSC markers [27]: For example, E/M and stemness markers have been found to be coexpressed in bladder cancer CTCs [28] and, accordingly, a single-cell transcription analysis of CTCs of brain lung cancer metastasis in the cerebrospinal fluids highlights the expression of CSCs markers, together with hybrid E/M markers [29]. The borderline between E/M plasticity and stemness become then evanescent, since the ability to acquire transient biological states within a “plasticity window” has been correlated with stemness [30]. The crucial involvement of EMT in metastatisazion is a recent matter of debate, due to the failure of lineage-tracing studies, moreover, evidences pointed out both the relevance of the epithelial phenotype in the establishment of metastasis, as well as the growing role of the hybrid E/M phenotype [31]. Conversely, the EMT involvement in drug resistance is strongly supported and univocally corroborated by preclinical and clinical findings (see below).

Other biological, stemness-related features can be transiently acquired thanks to the plastic properties of cancer cells: The ability to evade the immune system, the ability to adapt energy metabolism in different microenvironmental conditions (i.e., hypoxia), the ability to trans-differentiate to endothelial-like cells to provide metabolic support to hypoxic tumor regions (vasculogenic mimicry), as well as the ability to enter and exit a dormant state. Then, the “cancer stem cell” is now considered more a “state” than an “entity” [32]: Is the CSC plastic by definition, or is the plasticity of cancer cells helping to define a stemness phenotype? The plethora of biological mechanisms defining such phenotype comprises the involvement of signaling pathways resulting in the most challenging feature of CSCs: The escape from targeting strategies.

## 3. CSCs and Cytotoxic Therapies: Chemotherapy and Radiation Therapy

Cytotoxic therapies comprise a category of anticancer treatments aimed at inducing cell death in tumor cells. These treatment regimens can involve both the combination of the “classical” chemotherapeutic drugs and radiation therapies. Some chemotherapeutics, such as platinum-based drugs, anthracyclines or antimetabolites, share with radiotherapies the same mechanism of action, by means of direct DNA damage, whereas other chemotherapeutics, such as mitotic spindle poisons, exert their toxic effect through the inhibition of cell division by means of inhibiting microtubules dynamics. Cytotoxic therapies are currently used for the treatment of solid tumors and hematological malignancies in the absence of other first-line, effective and less toxic treatments. Beside the invasive off-target effects, chemotherapies and radiation therapies are associated with a mild, although not durable, response. Resistance to these treatment strategies has been correlated with CSCs, regardless the pre-existent or acquired stemness phenotype [33,34].

Genotoxic agents, such as platinum derivatives (cisplatin, oxaliplatin), anthracyclines (doxorubinin, daunorubicin, epirubicin), and radiation treatment are DNA-damaging options that trigger the DNA damage response (DDR), in which a sensitive cancer cell falls into cell cycle blockade followed by induction of the apoptotic cascade. These pathways involve DNA damage sensor proteins, such as ATM and ATR [35,36,37], that converge to the activation of the oncosuppressor protein p53, that in turn induces cell cycle blockade in G1 or G2/M phases, the activation of programmed cell death through the pro-apoptotic Bcl-2 family proteins, or eventually the induction of cell senescence/irreversible cell cycle arrest [38,39]. CSCs present a highly efficient DNA damage repair machinery, together with the overexpression of anti-apoptotic proteins such as Bcl-2 and Bcl-XL, rescuing the cell from the activation of cell death [40]. The repopulation of the tumor mass after genotoxic therapies is then promoted by the activation of signaling pathways associated with self-renewal and survival-related pathways, typical of CSCs (Notch, Wnt/β-catenin, Hedgehog, TGFβ, PI3K/Akt) [15,41,42,43]. Moreover, CSC-specific pathways have been directly involved in chemotherapy resistance. Indeed, growing evidences show the relevance of Wnt/β-catenin and PI3K/Akt in cisplatin resistance, as demonstrated by the fact that cisplatin sensitization might be achieved by repressing the PI3K/Akt/mTOR [44,45,46,47], as well as the Wnt/β-catenin signaling pathways [48,49,50,51]. It has been demonstrated that Zeb1, Ezh2 and BMI1, master regulators of the EMT process and, consequently, of the acquisition of stemness traits, are also correlated with the hyperactivation of ATM, responsible for double-strand breaks repair, as well as the constitutive activation of CHK1 or PARP1, in cell cultures and mouse models of breast cancer [52], CSC derived from colorectal cancer patients [53], and other cancer types [43]. In this context, CHK1 inhibitors have been shown to overcome prostate cancer chemoresistance in metastatic, castration resistant prostate cancer cell lines and xenografts [54]. The correlation between EMT and pathways related to DNA repair is an exquisite demonstration that the intrinsic EMT plasticity of cancer cells may promote their phenotypic switch toward a stem cell state, gaining adaptation and escape to genotoxic drugs [55]. Other studies support the involvement of the EMT process in chemotherapy resistance: The acquisition of mesenchymal traits through Snail induction has been related to cisplatin resistance in osteosarcoma cells and xenografts [56], the acquisition of cisplatin resistance in ovarian cancer cells has been related to EMT inducers like Twist [57] or miRNAs like mi-R10b [58], and similarly, doxorubicin resistance is acquired by breast cancer cells through Snail and Twist, that might be permissive for the upregulation of P-glycoprotein or through PARP1 upregulation [59].

In this context, high rates of tumor relapse/resistance after cisplatin treatment were related to CSCs in different tumors, including ovarian cancer, non-small cell lung cancer (NSCLC), colorectal cancer (CRC), liver cancer, head and neck cancer [55,60]. Similarly, radiotherapy resistance has been associated with CSCs in breast, colorectal, prostate, hepatocellular carcinomas [55,61,62,63,64]. Specifically, Bae et al. demonstrated through whole genome transcriptome analysis that irradiated colorectal carcinoma cells show a gene expression signature that resemble that of embryonic stem cells, with the overactivation of CSC-related pathways [63].

An additional mechanism of drug resistance implies that the selective pressure exerted by the cytotoxic drug may kill non-CSCs while sparing the CSC population, based on their different proliferation rate: Indeed, genotoxic molecules, radiotherapies, and mitotic spindle poisons, due to their molecular action modality, preferentially target rapidly-dividing cells. CSCs are slow-cycling by nature, unless considering their transient amplifying progeny that actively proliferates before commitment to differentiation [65], and are able to enter to the G0-quiescent cell cycle phase, that represents a reversible dormant state where the cancer cell is alive but not proliferating [66]. Again, the plasticity of cancer cells can promote the acquisition of a quiescent phenotype even in non-CSCs, and this process seems to be driven by cytotoxic therapies themselves [67]. The dormant state can be acquired by cancer cells not only following the exposure to drugs, but also in order to colonize and adapt to distant organs, and then give rise to metastasis (disseminated tumor cells, DTCs) [68]. One of the main players in the induction of a dormant phenotype within stressful conditions is the p38 MAPK, that through the activation of the p16-pRb pathway may promote cell cycle arrest [69,70,71] and is involved in dormancy and chemoresistance of head and neck, breast, esophageal, pancreatic carcinomas and cholangiocarcinoma, through the involvement of stemness-related genes such as Sox2 and Sox9 [69,72,73,74,75,76]. At the same time, senescent pathways shared with dormancy pathways (p38–p27–p21) can be activated by stemness promoters such as the TGFβ family members, even in the presence of high mitogenic signals, in prostate and breast cancer cells [77,78,79]. Importantly, other studies demonstrated the relevance of the EMT process in the acquisition of a dormant phenotype, thus contributing to chemotherapy resistance. A very recent paper from Francescangeli et al. demonstrated a link between cell quiescence and chemoresistance, both in CRC xenografts and patients. Particularly, slow-cycling CRC CSCs are characterized by high expression levels of the transcription factor Zeb2, together with a mesenchymal-like phenotype; moreover, Zeb2 has been demonstrated to be directly involved in the upregulation of the antiapoptotic factors pCRAF and pASK1, promoting chemoresistance and the further enrichment of the CSC population in mouse models. These findings are accompanied by the observation of a correlation between Zeb2 levels, quiescence and EMT genes upregulation and worse relapse-free survival in CRC patients [80].

The overexpression of detoxifying enzymes is another way by which CSCs can protect themselves from cytotoxic agents. In this context, aldehyde dehydrogenases class I (ALDH1) has been found to be overexpressed in CSCs and correlated with treatment resistance, within breast cancer [81,82,83], prostate cancer [84,85,86], colorectal cancer [87,88], lung cancer [89,90], ovarian cancer [91,92,93], and many others [94]. The contribution of ALDH1 to chemo- and radio-therapy resistance resides in its metabolic role within the tumor cell, that means the conversion of aldehyde groups into the less toxic carboxylate groups, thus metabolizing active compounds into less active molecules, as well as the maintenance of reactive oxygen species (ROS) at low levels, through a ROS-scavenging activity that is protective against oxidative stress [95]. Both these mechanisms lead to protection from DNA damage, providing the basis for the resistance to genotoxic agents. Moreover, it has been demonstrated that ALDH1 is able to promote the oxidation of retinaldehyde into retinoic acid, a well-known inducer of cancer cell stemness, further increasing malignancy traits [95,96].

Last but not least, the overexpression of multi-drug resistance (MDR) proteins is now considered a well-accepted marker of CSCs. The MDR proteins are efflux pumps belonging to the ATP-binding cassette (ABC) family, located to the plasma membrane, that are able to promote the extrusion of a wide variety of pharmacologically unrelated drugs, independently from their chemical structure or their mechanism of action. These receptors have been found to be overexpressed in CSCs of different tumors [97,98], from breast cancer (ABCB1, also called P-glycoprotein, P-gp; ABCG2) [99,100,101], to neuroblastoma (ABCG2, ABCA3) [98], lung cancer (ABCG2) [102,103], ovarian cancer (ABCG2, ABCB1) [104,105], melanoma (ABCB5, ABCG2) [106,107] [108], colon cancer (ABCG2) [109], osteosarcoma (ABCG2, ABCB1, ABCB5) [110]. The overexpression of these transporters may be promoted in non-CSCs by the direct exposure to the anticancer drugs, further confirming the plastic adaptation of cancer cells to hostile conditions [111]. Moreover, their overexpression is specifically regulated by stemness-related signaling such as pathways under the control of Myc, Oct4, Smoothened (SMO), and CD133 [112].

## 4. CSCs and Targeted Therapies

Targeted therapies comprise anticancer drugs, such as monoclonal antibodies or small synthetic molecules, that have been conceived in order to specifically target proteins involved in tumor progression; such proteins are uniquely present (i.e., mutated proteins), or at least overexpressed, in tumor cells, avoiding most of the off-target effects. Despite an excellent initial response rate, tumor relapse can occur due to the development of drug resistance. Similar to cytotoxic therapies, tumor regrowth can be promoted by the selection of resistant clones (primary resistance), or by the acquisition of resistance-related traits (secondary resistance). The first case implies the existence of a stem cell population able to: (1) maintain the heterogenicity of the tumor mass (with responder as well as non-responder clones) [113,114] and (2) survive to targeted therapies, repopulating the tumor mass [115]. On the other hand, secondary resistance implies the ability of cancer cells to activate alternative mechanisms in order to circumvent the inhibition of oncogenic signaling pathways, from the easiest induction of parallel pathways to the more complex phenotypic switch, both indicative of the importance of the plasticity of tumor cells [116]. Among targeted therapies, worth noting are those aimed at blocking cell proliferation or angiogenesis.

### 4.1. Targeting Proliferative Pathways

Proliferative pathways that are frequently overactivated, or aberrantly activated, in cancer cells are associated with the tyrosine kinase activity of membrane receptors (such as EGFR, HER2) or of cytoplasmic proteins (such as the MEK/ERK family).

EGFR tyrosine kinase inhibitors (TKIs, gefitinib, erlotinib, afatinib) are employed for those tumors in which this receptor is overexpressed or constitutively activated by mutations; however, cancer cells are able to escape such inhibition leading to tumor relapse. Non-small cell lung cancers (NSCLCs) carrying EGFR mutations are currently managed with TKIs, and tumor progression after treatment has been correlated with both genetic alterations and phenotypic transformations toward a mesenchymal phenotype [117]. Secondary genetic alterations include additional mutations on the EGFR gene [118], acquired mutations of MET [119,120] (or the overexpression of its ligand HGF) [121], and consequent overactivation of the PI3K/Akt downstream pathway. Other genetic alterations involve abnormal activation of KRAS and BRAF, or PTEN loss [117,122]. Conversely, in the absence of additional mutations, resistance to TKIs can be induced by the increased secretion of TGFβ1, a known EMT inducer, that in turn promotes the acquisition of a mesenchymal, drug-resistant phenotype [123]. Furthermore, the contribution of EMT to TKIs has been reported to be correlated to YAP/TEAD/Slug pathways, through an epigenetic reprogramming leading to a dormant state [124]. Whether the preexistence of resistant clones affects outcomes or the selective pressure of TKIs induces genetic alterations bringing to drug resistance is yet to be clarified [125]. However, lung cancer cells resistant to gefitinib show stemness features, as highlighted by studies performed both lung cancer cell lines and mouse models, and further confirmed by observations on patient-derived specimens [126,127,128].

Among 20% of breast cancers harbor HER2 overexpression. HER2 inhibitors (i.e., lapatinib, trastuzumab) are monoclonal antibodies developed in order to specifically target this receptor; however, around 60% of patients experience tumor progression [129]. It has been demonstrated that HER2 overexpressing cells have CSC traits, including drug resistance [130,131]. Furthermore, the receptor is directly involved in stemness maintenance and tumor-initiating potential, since HER2 overexpression in breast cancer cell lines induced CSC features in vitro and increased their tumorigenic potential in NOD/SCID mice [132]. Consequently, targeting HER2+ cells may be helpful in eliminating the CSC population, as demonstrated by preclinical and clinical findings [133,134,135]. How to explain tumor progression upon treatment? A vicious cycle is promoted between HER2 and the EMT capability of cancer cells: HER2 is able to promote stemness pathways, such as those related to TGFβ, Notch, Wnt/β-catenin, Hedgehog. Most of them are linked with the induction of a mesenchymal phenotype, through the upregulation of EMT-driving factors (ZEB1/2, Snail, Slug) with the consequent overexpression of EMT markers, like N-cadherin, vimentin and matrix proteinases. Metalloproteinases (MMPs, ADAMs) are in turn involved in the cleavage of transmembrane proteins involved in cell-cell and cell-matrix interactions, as well as membrane receptors. The cleavage of the HER2 extracellular domain results in a truncated and hyperactivated form that is unable to bind inhibitors, making the cell refractory to HER2-based therapies [136,137,138,139]. Moreover, EMT-driven trastuzumab resistance might be responsible of the acquisition of the triple-negative phenotype by HER2+/PTEN- breast cancer cells [140]. Additional studies showed that trastuzumab resistance might be promoted by factors involved in the upregulation of both Wnt/β-catenin and PI3K/Akt signaling pathways: Choi et al. reported that cyclin-dependent kinase 12 (CDK12), often amplificated concurrently with HER2, drives a CSC phenotype, along with a trastuzumab-resistant phenotype. In particular, CDK12 overexpression has been found to correlate with Wnt1 and Wnt3 expression, β-catenin activation, and consequently with increased expression of Wnt/β-catenin target genes, leading to the expansion of the CD44+/CD24-/ESA+ breast CSC population. These changes were paralleled by the persistent HER2 activation via a regulatory crosstalk with the ErbB/PI3K cascade, resulting in Akt phosphorylation, that in turn might support β-catenin activation via GSK3β phosphotylation [141]. These finding strongly support the relevance of Wnt and Akt pathways in drug resistance and CSC maintenance.

TKIs are successfully employed for the treatment of Chronic Myeloid Leukemia (CML). This hematological malignancy is specifically characterized by a unique chromosomal translocation within a hemopoietic stem cell, with the generation of a new chromosome, known as Philadelphia chromosome, originating from the fusion between the ABL1 oncogene (chromosome 9) and the BCR gene (chromosome 22). The resulting BCR-ABL1 fusion protein has an oncogenic role due to its enhanced tyrosine kinase activity, transforming the hemopoietic stem cell in a leukemic stem cell that is able to self-renew and give rise to a myeloproliferative malignancy [142]. Thus, the BCL-ABL1 protein represents the ideal candidate for the development of tumor-specific TKIs. Nowadays, different TKIs are available for CML treatment, with the third generation conceived to target the mutation responsible for the acquired resistance. However, the overall survival is still of 12 months [143,144]. In this context, leukemia stem cells (LSCs) have been demonstrated to persist and promote TKI resistance, suggesting that these cells might be not entirely dependent from this translocation for their survival. Indeed, several stemness-related pathways are related to their proliferation, quiescence and then survival after TKIs exposure. The antiproliferative activity of TKI has been linked with the modulation of FOXO target genes, inducing G1 cell cycle arrest [145]. However, BCL6, a downstream target of PI3K/Akt/FOXO pathway, has been involved in CML-initiating cell maintenance, since BCL6-dependent repression of Arf and p53 is required for colony formation in vitro, and tumor initiation in vivo. Accordingly, BCL6 targeting has been demonstrated to eradicate CML stem cells in patient-derived samples, thus highlighting the possibility to target BCL6 to circumvent CML stem cell-dependent TKI resistance [146]. Hedgehog signaling, as well as Wnt/β-catenin, Jak/STAT3 pathways, have been further hyperactivated upon TKIs treatment, promoting survival and quiescence [144,147,148,149,150]. Moreover, BCR-ABL1-positive cells are prone to genetic instability, with LSCs showing an increased tolerance to DNA damage and subsequent protection from apoptosis [142,144]. Importantly, during disease progression, the impairment of blast differentiation may occur, and this could be due to both BCR-ABL1 dependent and independent mechanisms, suggesting that the use of TKIs could be not sufficient to counteract the stemness potential, promoting the selection of an undifferentiated TKI-resistant clone [144].

Other proliferative pathways that rely on tyrosine-kinase function are related to the MAPK cascade. Mutations on proteins involved in such signaling mechanisms are key drivers of different types of tumor: B-Raf, N-Ras, K-Ras are found in melanoma, colorectal cancer, thyroid cancer and lung cancer, among others [151,152,153]. TKIs were developed in order to specifically inhibit the mutated proteins, i.e., vemurafenib is a small molecule specifically conceived in order to target the B-Raf V600E mutation [154]. These TKIs are associated with high initial response-rates; however, almost all patients experience drug resistance and tumor recurrence, mostly due to the downstream reactivation of the same proliferative pathway [155]. Other studies showed the activation of parallel proliferative pathways, promoting the survival of vemurafenib-resistant CSCs: for example, Prasetyanti et al. demonstrated that, in colorectal CSCs, this could be mediated by the overexpression of ErbB, mediating NRG-1β activation and Akt phosphorylation [156]. More recent findings demonstrated that the E/M phenotype switching could represent a relevant mechanism associated with TKI resistance, and extensive reports are now describing the strict connection between the phenotype switch toward a mesenchymal phenotype and CSCs in different tumors [155,157,158]. In particular, CSCs can exhibit two different phenotypes: One facing an epithelial, proliferative state, and another facing a mesenchymal, migratory state. The hybrid state, characterized by the expression of both epithelial and mesenchymal traits, has the highest plasticity/stemness potential, since it has the same potential to acquire different phenotypes [20]. Indeed, this state has been associated with circulating tumor cells (CTCs) responsible for distant colonization, and with drug resistance, being these cells as plastic enough to enter a dormant state, bypassing every attempt to target proliferative pathways [159].

### 4.2. Targeting Angiogenic Pathways

Antiangiogenetic drugs have been developed in order to inhibit the ability of cancer cells to drive the vascularization of the tumor mass, promoting tumor starvation. Proangiogenic factors secreted and/or overexpressed by tumor cells are VEGFs, that bind to their receptors (VEGFR1 and 2) on endothelial cells, or angiopoietins, that bind to the Tie2 receptor, so that targeted therapies comprise molecules specifically conceived to block these pathways (i.e., bevacizumab, trebananib). Since VEGFRs have a tyrosine kinase activity, these pathways can be additionally blocked with multikinase inhibitors (i.e., sorafenib) [160]. Additionally, the antiangiogenic drug everolimus is a TKI specifically targeting the mammalian target of rapamycin (mTOR) [161].

Different mechanisms have been associated with the development of antiangiogenic drug resistance, both primary and acquired [162]. Primary resistance can be due to the redundancy of angiogenetic pathways: several factors, such as EGFs, FGFs, TGFs, PDGFs, can contribute to the vascularization [162,163,164], compensating for VEGF blockade. One intriguing acquired mechanism exploited by cancer cells to escape antiangiogenic therapies is represented by the process of vasculogenic mimicry. It is described as the ability of cancer cells to shift their phenotype and behavior toward endothelial-like cells, so that they acquire the ability to form vascular-like structures to provide nutrients and oxygen to tumor cells. This phenomenon has been observed in different types of tumor (breast cancer, colon cancer, melanoma, ovarian cancer, glioblastoma, Ewing sarcoma among others) and has been associated with CSCs [165,166,167,168,169,170,171,172]. Accordingly, vasculogenic mimicry is correlated with CD133 and Lgr5 expression as well as tumor aggressiveness [173], even in patient-derived colorectal cancer specimens [174]; ALDH1+ and CD133+ breast cancer cells are able to form endothelial-like structure in matrigel culture [167,175]. CD133+ cells with cancer stem cell characteristics associate with vasculogenic mimicry, and a correlation has been observed between CD133+ and ALDH+ cells, vascular mimicry and tumor aggressiveness in ovarian cancer patients [176,177]; finally, ABCB5, CD133, CD271-positive melanoma cells have been found to be able to transdifferentiate to endothelial-like cells both in vitro and in vivo [178,179]. In this context, stemness-related signaling pathways are involved in vasculogenic mimicry. Wnt/β-catenin have been demonstrated to be involved in the acquisition of an endothelial-like phenotype in colon cancer [168], and Notch/Nodal signaling seems to be a crucial pathway involved in this process within a variety of malignancies such as melanoma, lung cancer, breast cancer [180,181,182,183,184]. Indeed, the inhibition of Notch impairs vasculogenic mimicry in melanoma and glioblastoma cells [185,186,187].

Being independent from the canonical angiogenetic signaling pathways, the formation of vascular-like structures is unaffected by targeted therapies, moreover, it might be elicited by the same antiangiogenic treatment [188]. One of the current theories is that antiangiogenic treatment itself generates hypoxia due to the inhibition of oxygen supply. As a rebound, the induction of HIF1α (Hypoxia Inducible Factor α), a known driver of EMT and stemness, promotes adaptation to oxygen deprivation driving the enrichment of the CSC population, consequent tumor regrowth and metastasis dissemination [189].

The key feature of tumor cells in shaping their phenotype is represented by cell plasticity. Again, the hybrid epithelial-mesenchymal state represents the highest plastic state of cancer cells, so that the EMT process has been strictly correlated both with stemness and with the potential to become an endothelial-like cell [184,190,191,192]. Accordingly, Twist1, one of the master regulators of the EMT process, has been found to be associated with vascular mimicry, through the upregulation of vascular-endothelial cadherins (VE-cadherins), in different tumors, such as breast cancer, hepatocellular carcinoma, colon cancer, so that the phenomenon can be called “epithelial-to-endothelial transition” [184,193,194,195].

## 5. CSCs and Immunotherapies

In recent years, the management of different types of tumors has taken advantage of the increasing knowledges within the immuno-oncology field. Immunotherapies have been developed in the last decades, with the aim to boost the immune system in the battle against cancer cells. The first immunotherapeutics were approved by the Food and Drug Administration (FDA) in 2011 (ipilimumab, anti-CTLA-4) and 2014 (nivolumab, anti-PD-1) for the treatment of unresectable metastatic melanoma [196,197,198], and subsequently for the treatment of other tumors [189]. Immune-checkpoint inhibitors (ICIs) counteract the inhibition exerted by tumor cells on professional antigen presenting cells (APCs) and effector, cytotoxic T cells (Teff). Indeed, the rationale underlying immunotherapeutic approaches is to favor antitumor immune responses, while counteracting the protumor immune environment. Cancer cells can acquire features promoting their immune-escape (i.e., through downregulation of tumor-associated antigens), the recruitment of pro-tumor immune cells (i.e., through the secretion of immunomodulatory cytokines), and the suppression of anti-tumor immune cells, through the secretion of immunosuppressive molecules and/or the overexpression of immune checkpoint regulators. In this context, the overexpression of immune-checkpoints such as PD-1 and CTLA-4 represents the basis for the development of specifically designed monoclonal antibodies. The overexpression of PD-1 or PD-L1 has been documented on CSCs within different tumor types, such as melanoma, ovarian cancer, breast cancer, colon cancer and lung cancer [19,199]. For example, PD-L1 has been found to be overexpressed in CSCs from breast and colon cancer cell lines [200]. Particularly, the analysis of triple-negative breast cancer human samples showed the presence of PD-L1-low and PD-L1-high cells, with the latter characterized by the concomitant overexpression of stemness pathways, and by a higher spheroidogenic and tumorigenic potential in vitro and in vivo, respectively [201]. Furthermore, the overexpression of PD-L1 on breast CSCs could be due to the hyperactivation of stemness-related pathways, since Wnt inhibitors were found to downregulate PD-L1 expression on breast cancer cell lines, whereas Wnt activators had the opposite effect [201]. As a potential analysis for lung cancer patient stratification before immunotherapy, PD-L1 positivity was shown on putative cancer stem cells from lymph node metastasis in patients aspirates [202]. Other data demonstrated that the cytokine-like factor transmembrane protein CMTM6, a direct regulator of the PD-1/PD-L1 axis through the stabilization of PD-L1 protein, has been recently involved in the upregulation of β-catenin activation and in the maintenance of a stemness phenotype in head and neck squamous cell carcinoma. Furthermore, CMTM6 has been implicated in the EMT promoted by TGFβ, in the overexpression of immune checkpoint molecules, and has been correlated with worse prognosis [203]. Additional supporting data regarding the strict relationship between stemness, EMT, immune-evasion and chemoresistance are provided by recent studies showing that immune-regulatory molecules such as CD200, PD-1, PD-L1 and CTLA-4 are directly involved in CSC maintenance and enrichment, activating the expression of the stemness-related genes Oct-4 and Nanog, promoting the EMT process, as well as the tumorigenicity and chemotherapy resistance of cancer cells [204,205,206]. The key role of EMT-related cell plasticity in immunosuppression and immunotherapy resistance has been highlighted in different tumor types [207], its prognostic value has been analyzed for gliomas [208], and TGFβ expression has been associated with poor prognosis in advanced bladder cancer, with a possible intrinsic resistance to PD-1 blockade [209]. Giving these observations, CSCs seem to represent an ideal target for ICIs. However, they have been involved in the development of immunotherapy resistance: in the absence of primary resistance, the excellent response and the enhanced survival rate can be subverted by the development of secondary, acquired, resistance, in almost 50% patients [210,211]. Beside the high expression of the immune checkpoint regulators, it has been demonstrated that the hyperactivation of the Wnt/β-catenin pathways is in turn responsible of an altered cytokine secretion by tumor cells, thus affecting the recruitment of dendritic cells and T cells and/or exerting a tolerogenic effect, in different types of tumor [212]. In this context, observations have been made about the correlation between the activation of the Wnt signaling and the absence of T cell infiltration in colorectal cancer patients [213]. Moreover, dendritic cells (DCs) tolerization has been demonstrated to be induced by Wnt5a signaling, via metabolic reprogramming in preclinical melanoma models [214]. Furthermore, the joint recruitment of immunosuppressive cells such as MDSCs (myeloid-derived suppressor cells) or regulatory T cells (Tregs) helps maintaining an immune-suppressive microenvironment, independently from PD-1/PD-L1 expression/blockade [189,211,215,216]. Other signaling pathways involved in immunotherapy resistance are related to TGFβ, a well-known inducer of stemness states, as well as of the EMT program. Indeed, this factor has been found to promote the differentiation of cytotoxic T cells into Treg cells, and to be correlated with ICI resistance [189,217]. As discussed above, the EMT has been extensively linked with stemness and general drug resistance. It is then not surprising that this process has also been correlated with refractoriness to immunotherapies. CD8+ T cells exhaustion can be triggered by molecules specifically involved in the EMT: the transcription factor ZEB1 and the micro-RNA miR-200 have been demonstrated to upregulate PD-L1 on cancer cells, thus leading to T cell suppression [218].

## 6. Targeting CSCs: A Challenge for Future Interventions

**Figure 1:**
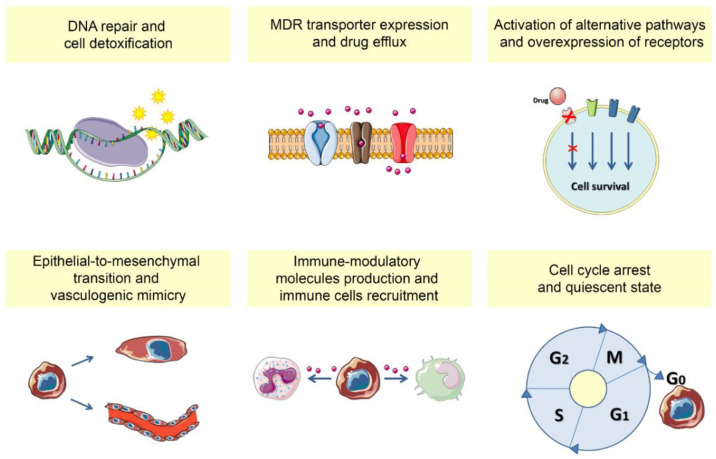
Molecular mechanisms helping CSCs in treatment-escaping strategies. DNA repair, cell detoxification, drug efflux, cell cycle arrest are mainly related to chemo-radiotherapy resistance. The escape from targeted, antiproliferative, therapies is mediated by the activation of alternative signaling pathways, whereas the escape from targeted, antiangiogenic therapies is mediated by vasculogenic mimicry. The ability to shape the immune contexture is related to resistance to immunotherapies.

Altogether, the molecular mechanisms described so far, and summarized in Figure 1, represent the basis for the acquisition of multiple drug resistance, leading to the clinical impact of CSCs, related to faster progression/metastatisazion and tumor relapse, thus tremendously affecting patient survival.

Indeed, the expression of CSC markers have been associated with significantly reduced overall survival in different tumors [3]. Several efforts have been made in the latest years to dissect the molecular features and understand the prosurvival pathways of CSCs, opening new ways for the development of innovative therapies. To date, different CSC-targeted therapies are under early clinical investigation. Moreover, few molecules specifically targeting CSC-related pathways have been recently approved for the treatment of different tumors (Table 1).

The first molecules to be approved by the FDA (vismodegib and sonidegib) are aimed at blocking the Hedgehog pathway, one of the master regulators of stemness properties. These drugs were introduced in the clinic for the treatment of locally advanced and metastatic basal cell carcinoma, and clinical evidence showed their efficacy also in pediatric and adult cases of recurrent/refractory medulloblastomas [219,220]. However, conflicting results have been obtained about the use of these molecules for the treatment of pancreatic, colorectal and other cancers, without significant benefits in terms of patient survival, with respect to standard chemotherapy [221].

CDK4/6 inhibitors (palbociclib and its analogues) have been approved for the treatment of hormone receptor (HR)-positive, HER2-negative, locally advanced or metastatic breast cancer, with the aim to block the self-renewal of CSCs. Clinical trials demonstrated their effectiveness when paired with standard therapies, doubling the median progression-free survival [222,223]. Importantly, due to their significant efficacy, CDK4/6 inhibitors are now recommended in the clinical practice, in the first-line or second-line setting in women with HR+, HER2- breast cancer, in combination with hormonal or targeted therapies [224].

Venetoclax is a BH3-mimetic drug, specifically targeting the antiapoptotic protein Bcl2. It was approved in 2016 for the treatment of chronic lymphocytic leukemia (CLL), and recently approved for the treatment of acute myeloid leukemia (AML) in combination with demethylating agents such as azacytidine [225]. As for its mechanism of action, this drug may potentially target two different features of CSCs, both related to mitochondria: 1. the overexpression of anti-apoptotic proteins, thus rendering CSCs susceptible to apoptosis-inducing drugs, and 2. the CSC dependance from oxidative phosphorylation, thus impairing their mitochondrial metabolism [221,225,226].

Beside the molecules that are already used in the clinical practice, other therapeutic approaches are under preclinical or early clinical investigation. Worth noting is the growing relevance of the tumor microenvironment in the definition of stemness features and CSCs niches. Then, an alternative approach aimed at targeting CSCs relies on the perturbation of this setting. For example, the overexpression of CXCR4 in leukemic stem cells is responsible of their bone marrow retention and quiescence, induced by CXCL12 secreted by stromal cells. Plerixafor, a CXCR4 antagonist approved to enhance the mobilization and collection of hematopoietic stem cells for autologous transplantation in patients with Non-Hodgkin’s lymphoma and multiple myeloma, is under investigation to be applied for the mobilization of leukemic stem cells, in order to improve their elimination from the bloodstream through chemotherapy. Promising results have been obtained in preclinical studies, and early clinical trials are ongoing [227]. Some early approaches are related to the inhibition of the classical stemness signaling pathways (i.e., Notch, Wnt, TGFβ) [221], whereas some others are conceptualized in order to specifically target CSC-related surface markers. Ideally, the development of monoclonal antibodies eventually conjugated with toxic compounds, as well as the development of CSC-specific CAR T cells, might selectively kill CSCs, however, the lack of specific, pan-CSC markers limits this approach in a broad setting [221,228]. However, although in a small subset of cancer types CSCs might be specifically characterized by the overexpression of defined proteins, these proteins could be potentially exploited for the development of anti-CSC vaccines. For example, very recent studies highlighted the relevance of MUC1 as an immunogenic protein, specifically overexpressed in colorectal cancer stem cells (CCSC). In these studies, mice immunization with CCSC lysates, followed by challenge with CT26 cells, significantly reduced tumor growth, and this was accompanied by a robust activation of an antitumor immune response. Furthermore, MUC1 overexpression in CD133+ CSCs significantly increased this effect, whereas MUC1 downregulation abrogated the effectiveness of this vaccine [229,230]. Similarly, following this approach, a MUC1-based autologous dendritic cell (DC) vaccine given to prostate cancer patients was reported to be safe in phase I/II studies and elicited a significant CD4+/CD8+ T cell response [231]. Similar findings have been observed with CD133 mRNA-loaded DC vaccination in humanized glioma models [232], CD44-loaded DCs in colorectal cancer [233], and different other approaches that might be based on DCs, T cells, and oncolytic viruses [234,235,236].

Beside to the few CSC-selective therapies that have been approved, and that can be defined successful so far, other unsuccessful approaches might be mentioned. A number of different promising candidates was shown to be ineffective in clinical trials, with slight or absent survival benefits: for example, the same Hedgehog inhibitors, that have been illustrated above, had modest activity only in a subset of tumors with active Hedgehog signaling [237]. Among other therapeutic failures, Notch inhibitors showed very limited activity, accompanied by off-target effects due to the physiologic relevance of this pathway (i.e., in the immune system regulation)[238]. In this context, the new redefined concept of stemness in terms of cell plasticity might play a pivotal role in this phenomenon. According to this point of view, and based on the considerations illustrated in this review, on one hand CSCs have the ability to adapt their phenotype to adverse conditions, on the other hand, every single cell in the tumor bulk, even the more differentiated cell, has the potential to regenerate the CSC population. However, novel interesting theories have been proposed, such as the coexistence of different CSC populations within the same tumor, with some being susceptible to treatment and some other being unresponsive, or the existence of more differentiated progenitor cells able to sustain the tumor itself [239].

## 7. Conclusions

CSCs play a key role in cancer relapse. This is due to their molecular advantages toward the escape from different therapeutic strategies, that have been illustrated in this review, and summarized in Figure 1. It is worth mention that the CSC phenotype, in relation with therapy resistance and tumor recurrence, is further finely regulated by the tumor-surrounding microenvironment. Indeed, different cell types (i.e., stromal cells, immune cells, endothelial cells) and different contextures (i.e., perivascular or bone marrow niches) can actively promote tumor cell plasticity, thus adding further complexity to the field (for extensive reviews, see [240,241,242,243,244].

Given the key role of CSC in therapy resistance and cancer relapse, CSC-targeting represents a fascinating and challenging approach aimed at cancer eradication. Several studies are ongoing, both at the clinical and preclinical level, for a broad range of novel chemical entities, as well as of innovative cellular- and biological-based therapies. However, the mechanisms underlying CSC plasticity are far to be fully understood, so that further unraveling of their molecular aspects might help in the development of more effective therapeutic strategies and in the achievement of durable response.

## Figures and Tables

**Table 1:** Novel therapeutic approaches targeting CSCs. Specific molecules have been recently approved for the treatment of breast cancer (CDK4/6 inhibitors), basal cell carcinoma (Hedgehog inhibitors), chronic lymphocytic leukemia and acute myeloid leukemia (Bcl2 inhibitors). Other CSC-targeted therapies are currently under clinical/preclinical investigation.

Drug/Agent	Molecular Target	Clinical Impact	Ref.
Vismodegib, sonidegib taladegib, glasdegib and itraconazole	Hedgehog inhibitors	Approved for treatment of basal cell carcinoma; Phase II/III clinical trials for treatment of medulloblastoma, prostate, pancreatic and hematological cancers	[166,167,168]
Palbociclib, ribociclib and abemaciclib	CDK4/6 inhibitors	Approved for treatment of HR+, HER2- breast cancer	[169,170,171]
Venetoclax	Bcl2 inhibitor	Approved for treatment of chronic lymphocytic leukemia and acute myeloid leukemia	[172]
Plerixafor	CXCR4 antagonist	Phase II clinical trials for treatment of acute myeloid leukemia	[174]
MK-0752, RO4929097 and PF-03084014; demcizumab	Notch inhibitors	Phase I/II clinical trials for treatment of glioma, NSCLC, breast and pancreatic cancer, T-cell acute lymphoblastic leukaemia	[168]
OMP-54F28; PRI-724 and CWP232291	Wnt inhibitors	Phase I clinical trials for treatment of pancreatic cancer, myeloma and acute myeloid leukemia	[168]
Galunisertib; fresolimumab and trabedersen	TGFβ inhibitors	Phase II clinical trials for treatment of glioblastoma, unresectable HCC mesothelioma, melanoma, and RCC glioma, pancreatic cancer	[168]
Catumaxomab	Anti-EpCAM antibody	Phase III clinical trials for treatment of advanced epithelial tumors and malignant ascites	[168]
SL-401, SGN-123A, talacotuzumab, MGD006, KHK2823, CAR-T	Anti-CD123 antibodies	Phase II clinical trials for treatment of hematological cancers	[168]
TTI-621	Anti-CD47 antibody	Phase I/II clinical trials for treatment of hematological cancers	[168]
Dofequidar	MDR inhibitor	Phase III clinical trials for treatment of breast cancer	[168]
Tn-MUC1 vaccine	Anti-MUC1 vaccine	Phase I/II trials for treatment of prostate cancer	[178]

## Data Availability

Not applicable.
